# Surgical planning in patients with brain glioma using diffusion tensor MR imaging and tractography

**DOI:** 10.1186/s43055-021-00490-5

**Published:** 2021-04-16

**Authors:** Mohamed E. Shalan, Ahmed Y. Soliman, Ibrahim A. Nassar, Reda A. Alarabawy

**Affiliations:** 1 M.B.B.CH ,Faculty of Medicine, Tanta University, Zefta, Gharbiya Egypt; 2 Neurosurgery ,Faculty of Medicine, Tanta University, Zefta, Gharbiya Egypt; 3 Radio-diagnosis and medical imaging Department, Faculty of Medicine, Tanta University, Zefta, Gharbiya Egypt

**Keywords:** Surgical planning, Diffusion tensor, Gliomas, Magnetic resonance imaging, Tractography

## Abstract

**Background:**

Diffusion-tensor imaging (DTI) is a magnetic resonance imaging (MRI) technique that depicts the integrity of white matter (WM) tracts. This study was conducted to assess the utility of DTI tractography as an imaging technique in assessment of brain gliomas and planning of the surgical corridor.

**Results:**

Twenty adult patients with brain gliomas were included. Neurological examination and conventional MRI and DTI scans were performed before and after surgery. Low-grade and high-grade tumors were found in 30% and 70% of patients, respectively. Preoperative DTI demonstrated five patterns of WM tract involvement: non-affected (10%), displaced (75%), edematous (55%), infiltrated (60%), and disrupted (20%). The obtained DTI scans were used for preoperative planning of the surgical corridor and extent of resection to achieve the maximum resection while preserving the WM tracts. Total resection was achieved in 40%, while 60% underwent subtotal resection. Postoperative neurological examination showed deterioration of cognitive function, motor power, and vision in 15%, 10%, and 5% of patients, respectively. Headache persisted in 15%, while motor power improved in 35% of patients. High-grade tumors were significantly associated with higher percentage of subtotal resection (*p*=0.018) and pattern IV (*p*=0.018). There was a significant association between the preoperative pattern of WM tract involvement and the postoperative DTI changes (*p*<0.001).

**Conclusion:**

DTI enables assessment of displaced and infiltrated WM tracts in the vicinity of brain tumors. Preoperative planning of tumor resection and surgical corridor should include DTI scan to achieve the balance between maximum resection of tumor and maximal preservation of function.

## Background

Intracranial neoplasms may invade both functional cortical gray and white matter (WM) tracts. The goals of surgery in such patients include maximizing tumor resection and the preservation of vital cerebral functions. On one hand, total resection of the tumor reduces the risk of recurrence and increases the likelihood of adjuvant radiotherapy or chemotherapy to be more effective. On the other hand, sparing functionally relevant areas will preserve motor, language, or visual functions and hence significantly improves the patients’ quality of life [1, 2].

These considerations mandate a comprehensive assessment of the extent of the tumor before initiation of treatment. Magnetic resonance imaging (MRI) serves a pivotal role in diagnosing a variety of brain pathologies, including neoplasms. Brain neoplasms can be readily detected using conventional MRI. Enhanced MRI uses contrast medium to show the margins of the tumors as well as their different enhancement patterns, which can help indicate the tumor stage and, to some extent, tumor pathology. Functional MRI is a relatively new imaging modality that can show the functional areas in gray matter by applying specific task/stimulation related to each cortex [3–5].

However, these MRI modalities do not give precise information on WM tracts that may be involved in brain tumors. Diffusion tensor imaging (DTI) and tractography are MRI techniques that permit the depiction of WM by tracking the diffusion of water molecules [6]. The WM tracking is based on the direction of fibers and the anatomical location. Color-coded maps are generated from the tensor asset images and each WM fiber tract is assigned a specific color based on the direction of its fibers. Consequently, the use of DTI and tractography can confirm the integrity of WM tracts and localize a markedly deviated tract. Preoperative DTI and tractography are highly useful in planning resection [7].

The aim of this study was to assess the utility of DTI tractography as an imaging technique in pre- and post-surgical assessment of brain gliomas and planning of the surgical corridor, in order to cause the least damage to the WM tracts and obtain favorable functional outcomes.

## Methods

### Ethical considerations

The study was conducted following approval from the Research Ethics Committee, Faculty of Medicine in our university . Written consent was obtained from each patient after receiving full information about the study aim and methods. Confidentiality of patients’ data was maintained by assigning a code number to each patient that was known only by the researchers and the datasheet was kept anonymous.

### Study design and setting

This prospective study was carried out on patients with brain gliomas attending the Diagnostic Radiology Department at our University Hospitals between February 2019 and October 2020.

### Inclusion criteria

We included adult patients (of either gender) who were radiologically suspected to have brain gliomas attending the diagnostic Radiology & neurosurgery departments

### Exclusion criteria

We excluded patients who have contraindication to perform MRI, such as those with intraocular metallic foreign body, cardiac pacemaker, or non-compatible intracranial clips of arterial brain aneurysms.

### Procedures

All included patients were subjected to history taking, thorough clinical examination, and MR imaging with DTI.

History taking included the age, gender, and clinical presentation. Clinical examination included general assessment of the patients besides neurological examination (mental state, speech, cranial nerves, motor system, reflexes, coordination, involuntary movement, sensory system, sphincter disturbances, and gait disturbances).

Preoperative and postoperative MR imaging was performed at 1.5 Tesla unit (GE signa explorer closed magnet 1.5 T) using a standard quadrature birdcage head coil. The sequences used were sagittal T1-weighted image, sagittal T2-weighted image, 3D sequences, DTI, and tractography. The DTI and 3D T1 Fast Field Echo images were transferred to the offline workstation where the DTI images underwent linear registration and were opened in the MR diffusion application for production of fractional anisotropy maps (FA; measure of directionality of diffusion in a given voxel), color maps, apparent diffusion coefficient (ADC), and diffusion-weighted imaging trace. Registered diffusion tensor images were then loaded into the fiber-tracking application, and the 3D T1 Turbo field echo images were used as the anatomical underlay for better localization of seed placement locations.

Color-coded FA maps were viewed with anatomical images underneath, giving both anatomical information and information about fiber orientation. A specific color was assigned to tracts running in the three orthogonal planes: red for side-to-side tracts, green for anteroposterior tracts, and blue for craniocaudal tracts.

The anatomical tracts that were tracked included corticospinal tract, superior longitudinal fasciculus, inferior longitudinal fasciculus, corpus callosum, and uncinate fasciculus (Table 1).

**Table 1:** Method of tracking the white matter fiber tracts

Tract	Method of tracking
**Corticospinal tract**	Three ROIs were placed on transverse color-coded diffusion tensor images: in the pons anteriorly, in the posterior limb of internal capsule and at the motor cortex (precentral gyrus)
**Superior longitudinal fasciculus**	Two ROIs were placed in the cerebral deep WM on a coronal directional color-coded map: an anterior ROI in the plane passing through the reconstructed corticospinal tract and a posterior ROI in the plane passing through rostral surface of the splenium of the corpus callosum
**Inferior longitudinal fasciculus**	Two ROIs: the first was at the parieto-occipital sulcus (which was identified at the middle of the coronal section along the superior inferior axis) and the second was on a coronal section at the mid-temporal lobe at the section level of the posterior tip of the putamen
**Corpus callosum**	The primary ROI was placed in the corpus callosum in the mid sagittal plane. Secondary ROIs were placed, with two ROIs on the coronal color-coded map and two ROIs on the transverse color-coded map. Anterior callosal fibers were reconstructed by placing the ROI covering the deep WM in the coronal plane anterior to the genu of the corpus callosum. The posterior callosal fibers were reconstructed by placing the ROI posterior to the splenium of the corpus callosum. Callosal body fibers were reconstructed by placing the ROI at the centrum semiovale in the transverse plane superior to the body of the corpus callosum. The temporal interhemispheric connection fibers were reconstructed by placing ROIs bilaterally in the temporal deep WM, lateral to the trigone of the lateral ventricles. These four fibers were combined to delineate the entire corpus callosum.
**Uncinate fasciculus**	The seed area in the WM of the frontal lobe was located on the coronal planes at the tip of the frontal horn of the lateral ventricle, and the target area in white matter was located on the coronal planes at the tip of the inferior horn of the lateral ventricle in the ipsilateral temporal tip

*ROI* region of interest, *WM* white matter

The gliomas were graded according to the 2016 WHO grading system [8] in which pathologies are classified into four grades, with higher grades implying lesser degrees of differentiation, increasing anaplasia, increasing proliferative potential, and mitotic activity.

The effect of brain tumors on white matter tracts was classified into five patterns as follows: pattern I (not affected; fibers are in the correct anatomical location, characterized by normal FA and normal ADC similar to the homologous tract in contralateral hemisphere), pattern II (displacement; characterized by normal or mildly decreased FA and normal or mildly increased ADC relative to the homologous tract in contralateral hemisphere, with abnormal location and/or direction resulting from bulk mass displacement), pattern III (edematous; characterized by decreased FA and increased ADC. The tract appeared in place or deviated on directional color maps with same color but fainter due to decreased FA), pattern IV (infiltrated and partially disrupted; characterized by substantially decreased FA and increased ADC. The infiltrated tract is in place or deviated on directional color maps with abnormal hues mostly attributed to disrupted fibers extending into different directions), and pattern V (disrupted; characterized by isotropic or near-isotropic diffusion, such that the tract or part of it was not identifiable on FA or directional color maps) [9, 10].

With the assistance of the information provided by MRI, DTI, and WM tractography, we knew the relationship between the lesion and important WM tracts. Individualized surgical approaches were designed according to the information provided by the DTI and conventional MRI and the surgery was then performed accordingly. Postoperative detailed imaging were performed 4 months after operation to look for the extent of resection and any surgical complication and neurological outcome.

### Statistical analysis

Statistical analysis was performed using Statistical Package for Social Sciences (IBM SPSS Statistics) for Windows, version 26 (IBM Corp., Armonk, NY, USA). Qualitative variables were summarized as frequencies (count and percentage). Fisher’s exact test (for 2 × 2 contingency tables) or Fisher-Freeman-Halton exact test (for *r* × *c* tables) were used to examine the association between two categorical variables. A *p* value <0.05 was chosen to interpret tests of significance.

## Results

**Fig. 1:**
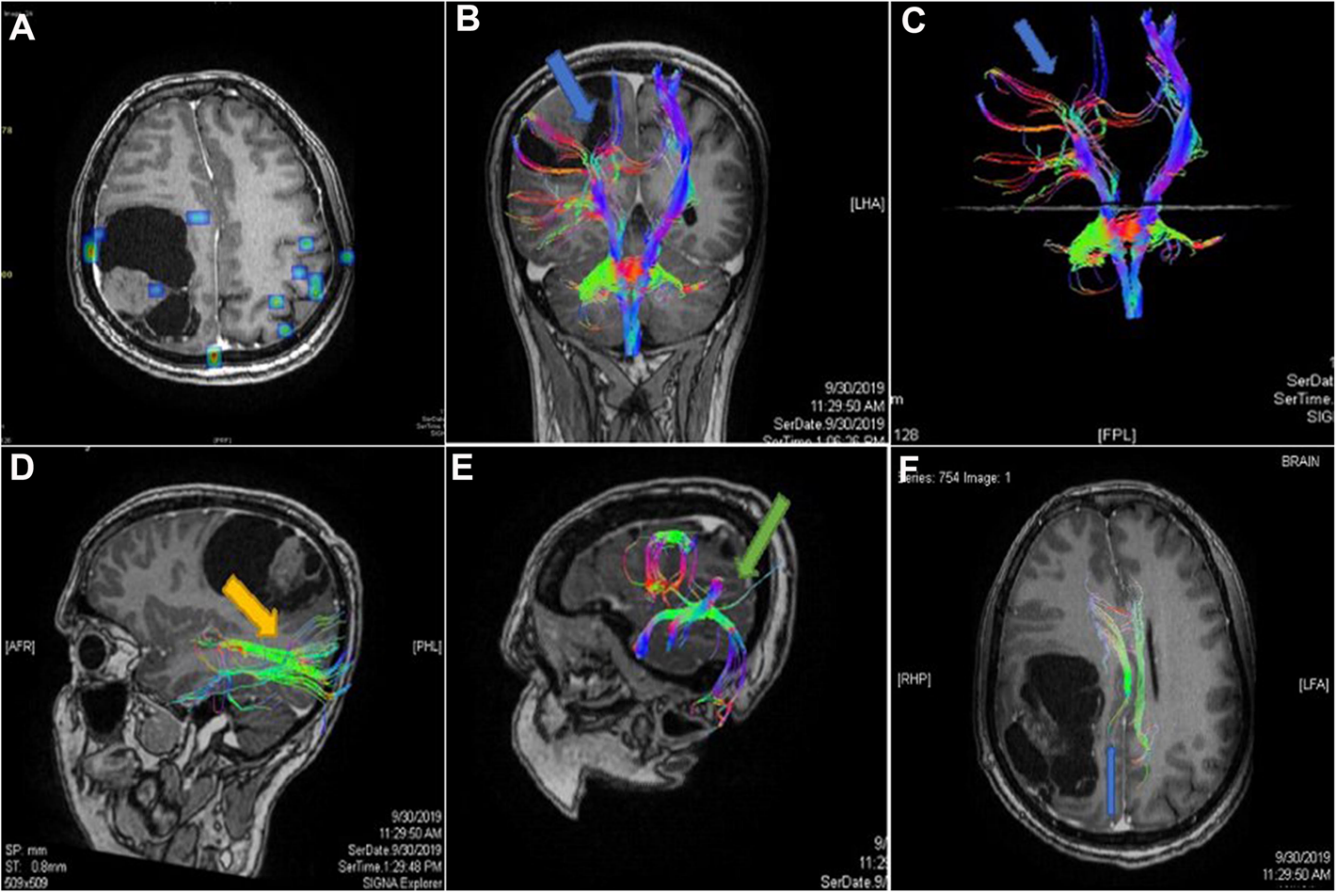
Case number 1. A 53-year-old woman with a right parietal anaplastic astrocytoma, preoperative radiological examination. **a** Axial T1 WI with contrast revealing a right parietal lobe space occupying lesion with faint heterogeneous post-contrast enhancement. **b**, **c** Coronal T1 WI with 3D reconstruction of the corticospinal tracts (CST) showing pattern IV outward displacement, splaying and distal fibers destruction of the right CST (Blue arrows). **d** Sagittal T1 WI with 3D reconstruction of the right inferior longitudinal fasciculus (ILF) tract showing pattern II attenuation of ILF (orange arrow). **e** Sagittal T1 WI with 3D reconstruction of the right superior longitudinal fasciculus (SLF) showing pattern VI displacement and attenuation of the right SLF (green arrow). **f** Axial T1 WI with 3D reconstruction of the cingulum tracts showing pattern III attenuation of the right tract (blue arrow)

**Fig. 2:**
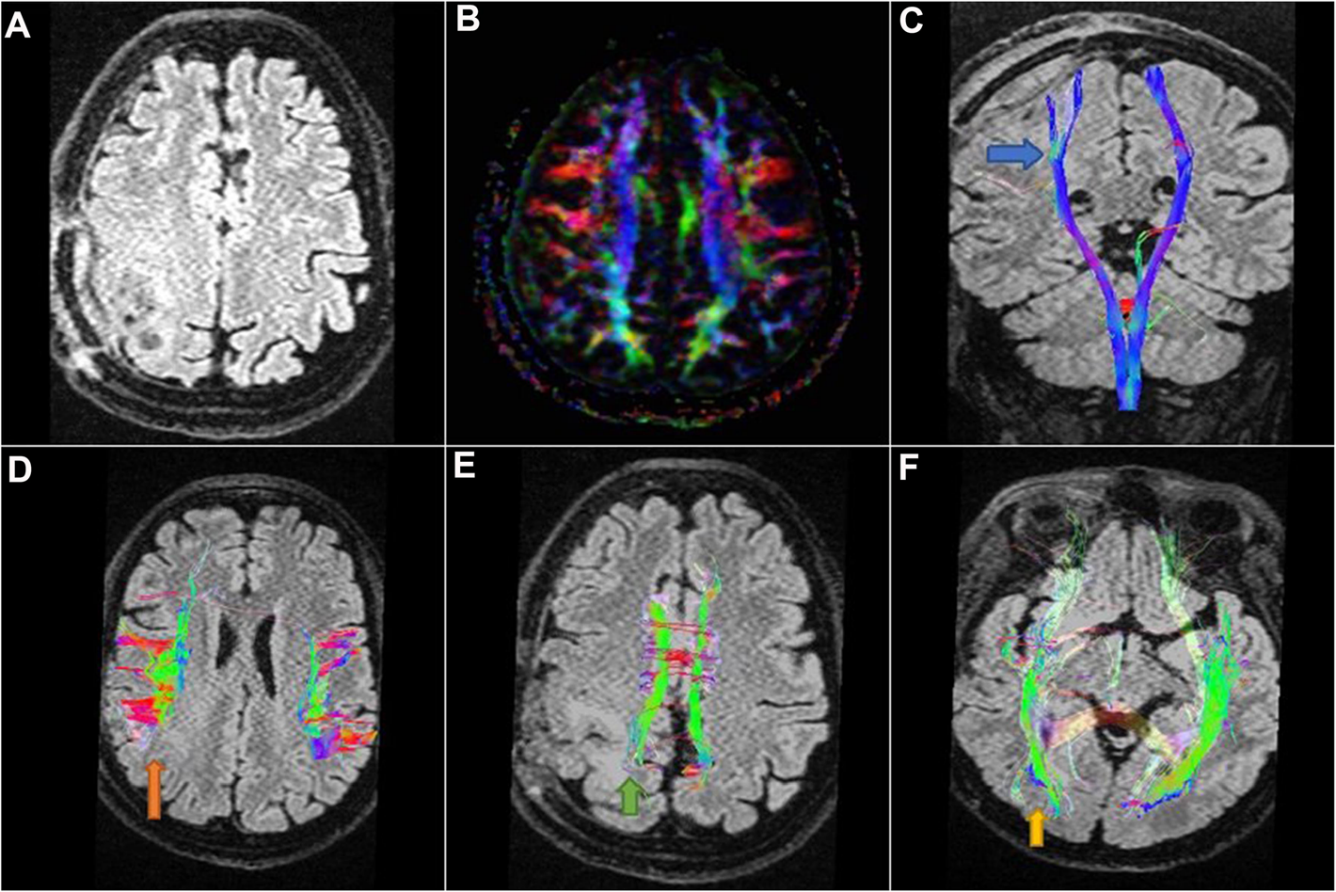
Case number 1. Postoperative radiological examination of Fig. 1 patient. **a**, **b** Axial FLAIR WI and DTI color-coded map revealing right parieto-occipital craniotomy flap with right parietal postoperative changes, no residual masses. **c** Sagittal FLAIR WI with 3D reconstruction of the corticospinal tracts revealing normalization of position and anisotropy of the splayed tracts (blue arrow). **d** Axial FLAIR WI with 3D reconstruction of superior longitudinal fasciculus (orange arrow). **e** Axial FLAIR WI with 3D reconstruction of the cingulum (green arrow) revealing normalization of anisotropy. **f** Axial FLAIR WI of inferior longitudinal fasciculus (yellow arrow) revealing normalization of position and anisotropy of their tracts

**Fig. 3:**
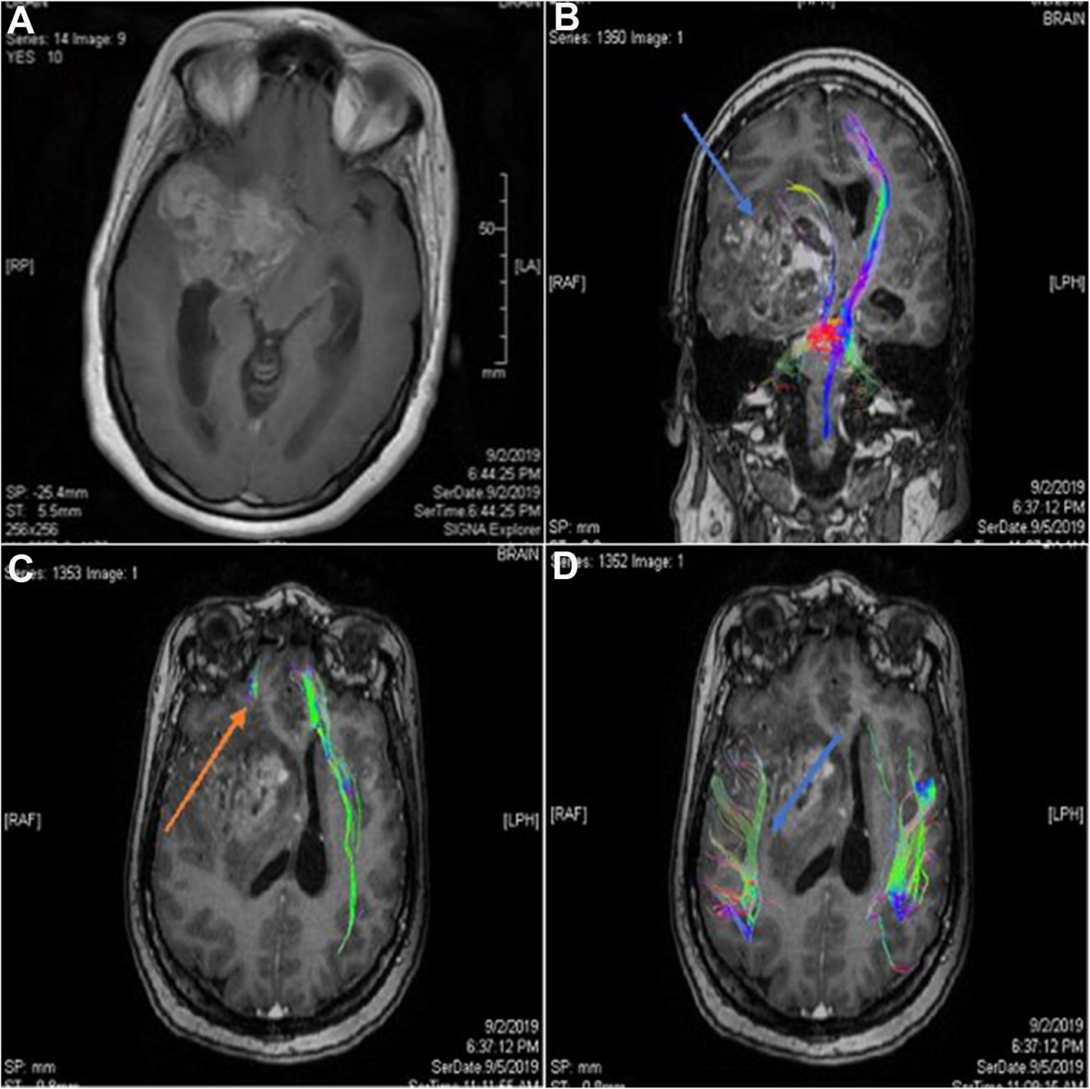
Case number 2. A 42-year-old woman with a right temporo-frontal, high-grade gliosarcoma, preoperative radiological examination. **a** Axial T1 WI with contrast revealing heterogeneous post-contrast enhancement surrounded by grade II vasogenic edema. **b** Coronal T1 WI with 3D reconstruction of the corticospinal (CST) tracts showing pattern IV inward displacement, attenuation, and fiber destructions of the right CST (blue arrow) compared to intact left CST. **c** Sagittal T1 WI with 3D reconstruction of the inferior longitudinal fasciculus (ILF) and part of uncinate fasciculus tracts showing pattern V destruction of the tracts on right side (orange arrow). **d** Sagittal T1 WI with 3D reconstruction of the superior longitudinal fasciculus (SLF; blue arrow) showing pattern II mild decreased FA, yet no fibers displacement on the right side

**Fig. 4:**
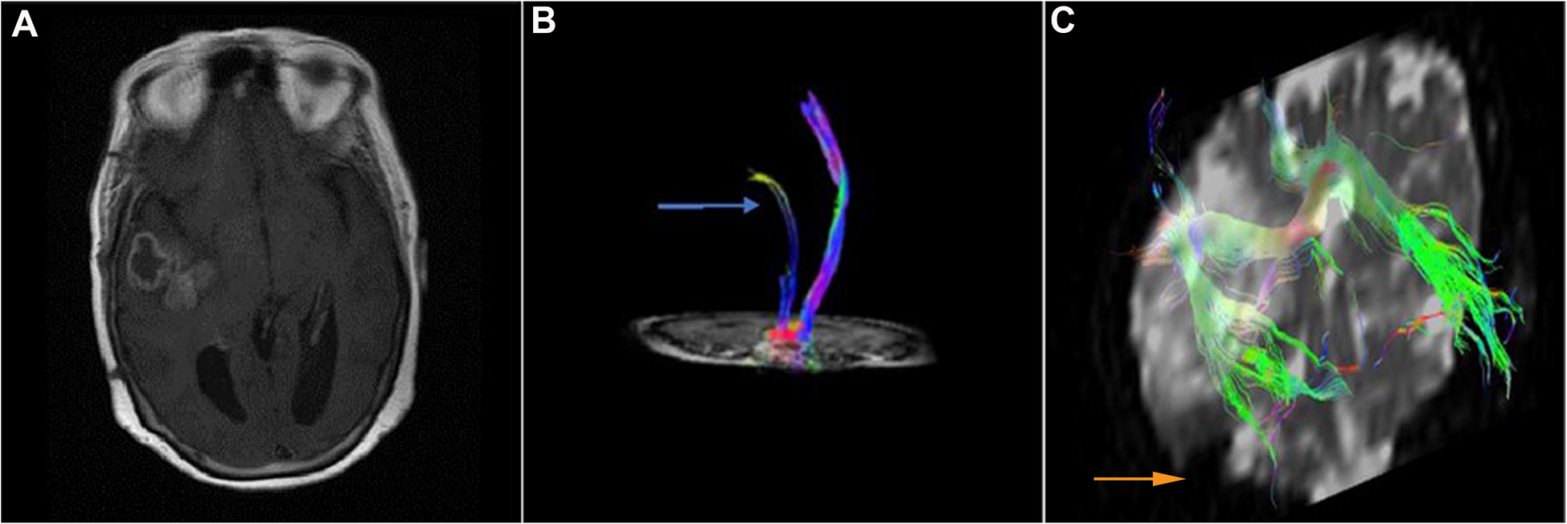
Case number 2. Postoperative radiological examination of Fig. 3 patient. **a** Axial T1 WI with contrast revealing a small residual. **b** Coronal view with 3D reconstruction of the CST (blue arrow) shows no fiber position or fraction anisotropy improvement. **c** Coronal view with 3D reconstruction of the SLF tracts (orange arrow) revealing normalization of the position and anisotropy

**Fig. 5:**
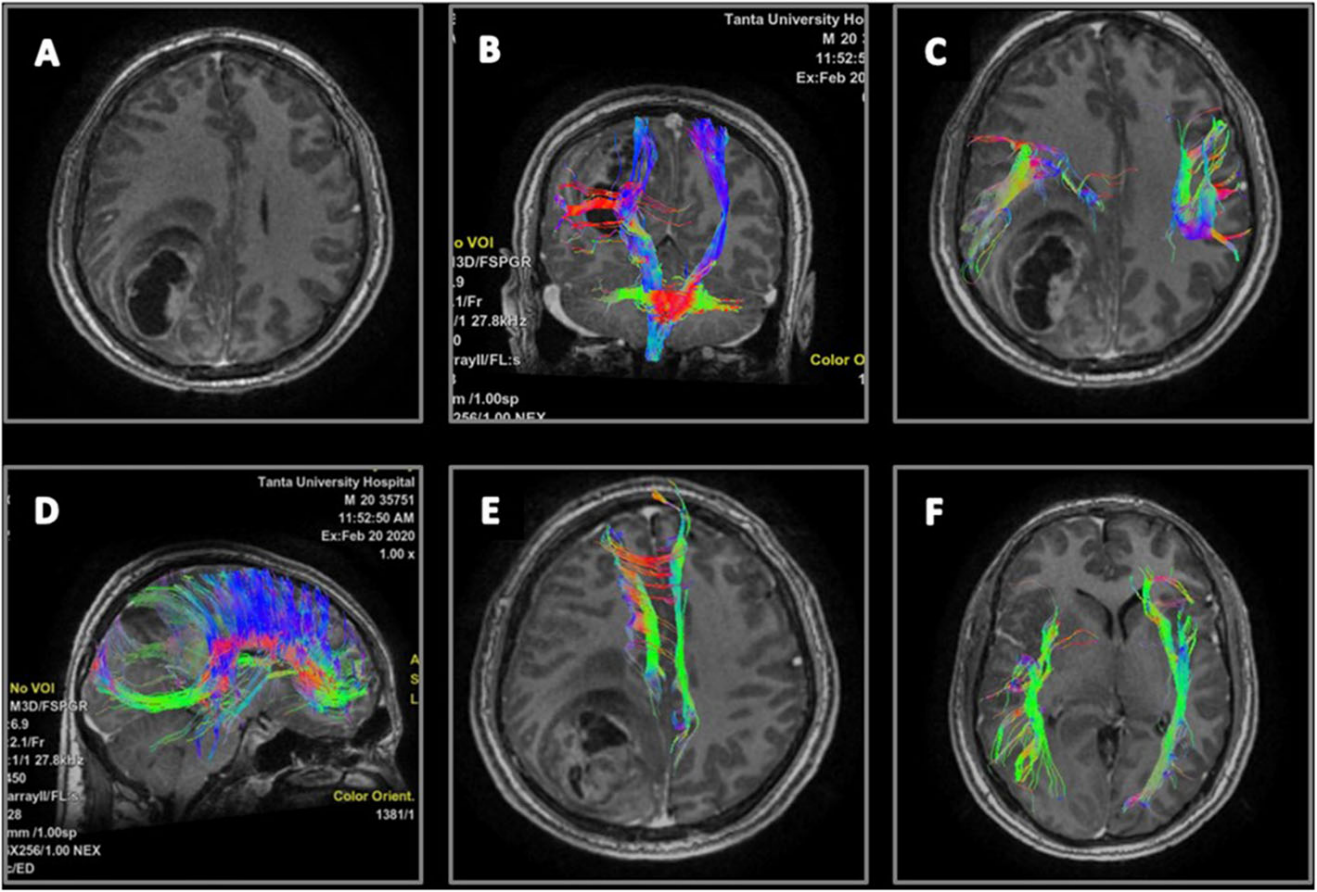
Case number 3. A 20 years old male patient presenting with headache and left side hemiparesis. Pathologically proven glioblastoma, high grade. **a** Axial T1 WI with contrast revealing a right parieto-occipital mixed solid and cystic SOL with faint heterogeneous post contrast enhancement surrounded by grade I vasogenic edema. **b** Coronal T1 WI with 3D reconstruction of the CST tracts. Pattern VI displacement, splaying, and fiber attenuation of the right CST related to the tumor compared to intact left CST. **c** Axial T1 WI with 3D reconstruction of the SLF. Pattern II antero-lateral displacement and mild decreased FA of the right SLF. Axial T1 WI with 3D reconstruction of **d** the corpus callosum tracts, **e** the cingulum tracts, and **f** the ILF tracts revealing pattern II displacement and splaying of CC shows mild decreased FA and mild increased ADC. Pattern III fibers attenuation of right cingulum and right ILF showing mild decreased FA and mild increased ADC

**Fig. 6:**
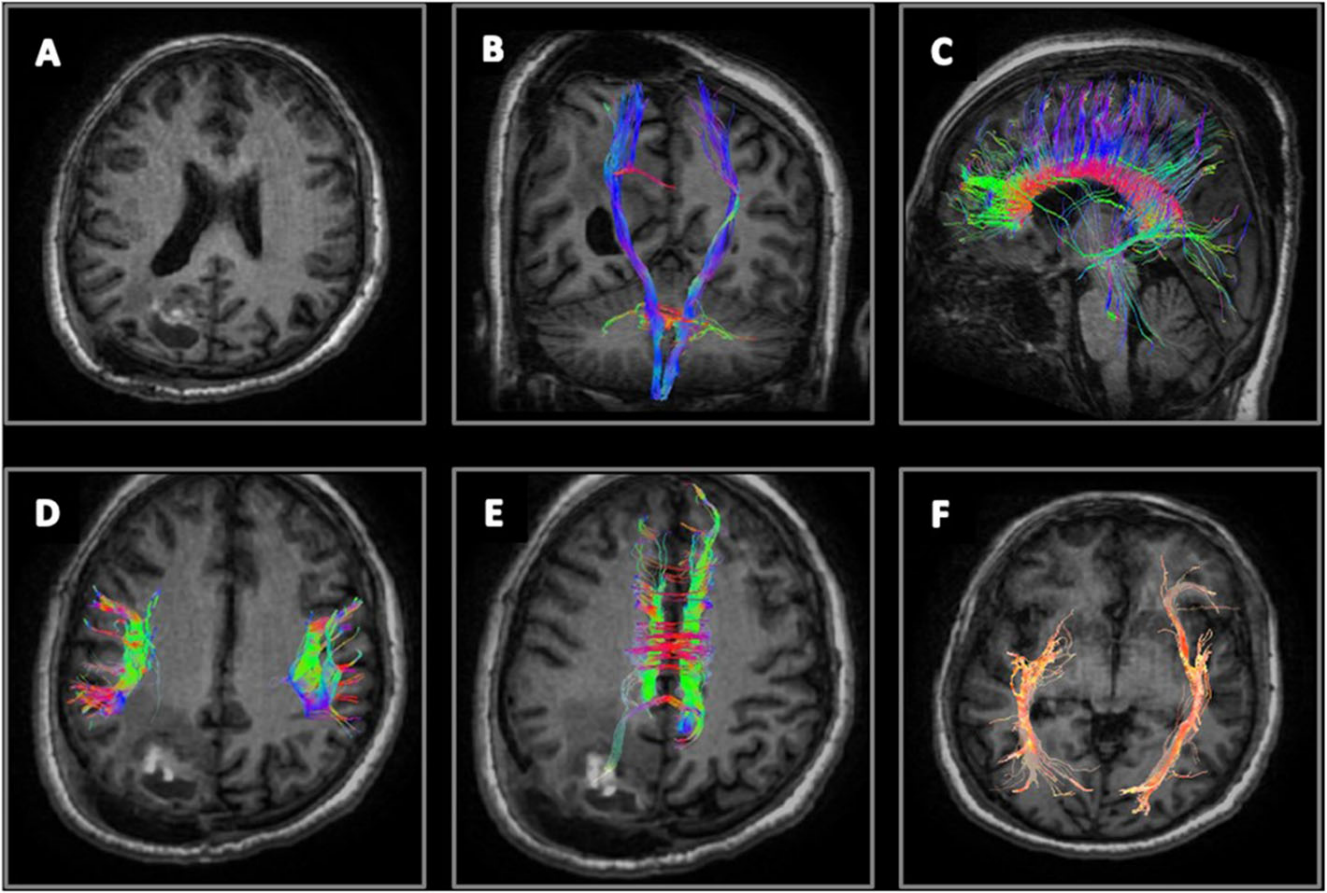
Case number 3. **a** Postoperative axial T1 WI revealing a small right parieto-occipital residual with small hemorrhagic component surrounded by grade I vasogenic edema. **b** Coronal T1 WI with 3D reconstruction of the CST tracts revealing normalization of position and anisotropy of the intact right CST. **c** Sagittal T1 WI with 3D reconstruction of the CC tracts. **d** Axial T1 WI with 3D reconstruction of SLF revealing normalization of position and anisotropy. **e** Axial T1 WI with 3D reconstruction of the cingulum. **f** Axial T1 WI with 3D reconstruction of the ILF revealing normalization anisotropy

**Fig. 7:**
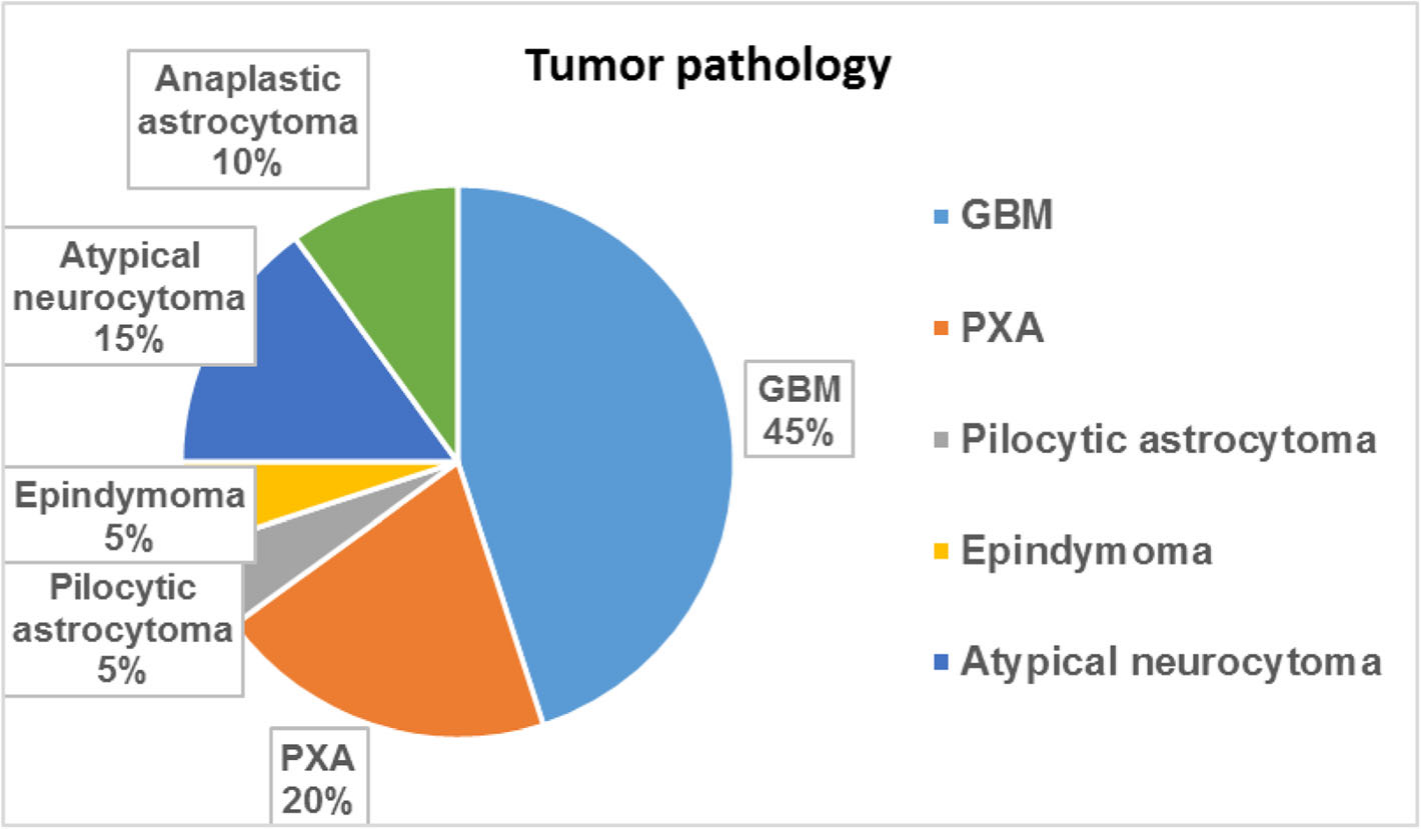
Pathology of brain tumors

Twenty patients were included in this study comprising 14 males (70%) and 6 females (30%). Their ages ranged from 20 to 55 years (Figs. 1, 2, 3, 4, 5, and 6). Among the 20 studied cases, 15 patients (75%) complained from headache, 9 patients (45%) were suffering from one-sided weakness (hemiplegia or hemiparesis), 7 patients (35%) had cerebral dysfunction (disturbed consciousness level, generalized convulsions and syncope), 4 patients (20%) had visual complaints (diminution of vision or diplopia), and 1 patient (5%) had behavioral changes. Our study included different types and grades of brain glioma (Table 2, Fig. 7).

**Table 2:** Patients’ and tumors characteristics (total *n* = 20)

Parameter	No	%
**Age (Years)**		
≤ 30	3	15.0
˃ 30–40	5	25.0
˃ 40–50	8	40.0
˃ 50	4	20.0
**Gender**		
Male	14	70.0
Female	6	30.0
**Complaint**		
Headache	15	75.0
One-sided weakness	9	45.0
Cerebral dysfunction	7	35.0
Visual complaints	4	20.0
Behavioral changes	1	5.0
**Tumor grades**		
Grade I	1	5.0
Grade II	5	25.0
Grade III	5	25.0
Grade VI	9	45.0
**Affected region/lobe**		
Parietal	8	40.0
Temporal	6	30.0
Occipital	2	10.0
Frontal	4	20.0
**Tumor patterns**		
**I**	2	10.0
**II**	15	75.0
**III**	11	55.0
**IV** **V**	12 4	60.0 20.0

Preoperative conventional MRI demonstrated that 14 patients (70%) had tumors in the parieto-temporal region, 12 patients (30%) in the occipital region, 8 patients (20%) in the frontal region, and 2 patients (5%) had tumors invading and crossing midline through the corpus callosum. Tumors were classified into two main groups: low-grade (grade I and II lesions, 6 patients) and high-grade (grade III and IV lesions, 14 patients) groups (Table 2).

Preoperative DTI revealed the effect of brain tumors on WM tracts which was classified into five patterns: non-affected (10% of cases), displacement (75%), edematous (55%), infiltration (60%), and disruption (20%) patterns (Table 2). Combined pattern of affection has been detected in most cases.

Surgical corridor and the extent of surgical excision was planned according to the affection of WM fiber tracts as detected by DTI, taking into consideration the balance between maximum resection and preservation of WM tracts. Accordingly, gross total resection was achieved in 8 (40%) patients, while 12 (60%) patients underwent subtotal resection of tumors. Postoperative neurological examination during follow-up showed that cognitive function, motor power, and vision deteriorated after surgery in 3 (15%), 2 (10%), and 1 (5%) patients, respectively. Headache persisted in 3 (15%) patients. Improvement in motor power was observed in 7 (35%) patients (Table 3).

**Table 3:** Association between tumor grade and each of surgical excision and outcome (total *n* = 20)

Parameter	High		Low		Total		***p***^**a**^
	No	%	No	%	No	%	
**Surgical excision**							
Subtotal	11	78.6	1	16.7	12	60.0	0.018*
Total	3	21.4	5	83.3	8	40.0	
**Postoperative outcome**							
Deteriorated cerebral function	3	21.4	0	0.0	3	15.0	0.521
Deteriorated motor power	2	14.3	0	0.0	2	10.0	1.000
Deteriorated visual complaint	1	7.1	0	0.0	1	5.0	1.000
Headache	2	14.3	1	16.7	3	15.0	1.000
Improved motor power	5	35.7	2	33.3	7	35.0	1.000
**Pre- and postoperative imaging**							
**Preoperative pattern I (not affected)**	0	0.0	2	33.3	2	10.0	0.079
Normalized position and anisotropy	0	0.0	2	33.3	2	10.0	
**Preoperative pattern II (displacement)**	10	71.4	5	83.3	15	75.0	1.000
**Postoperative**							
Normalized position and anisotropy	7	70.0	5	100.0	12	80.0	0.505
Normalized position and partial FA improvement	3	30.0	0	0.0	3	20.0	
**Preoperative pattern III (edematous)**	7	50.0	4	66.7	11	55.0	0.642
**Postoperative**							
Normalized position and anisotropy	4	57.1	3	75.0	7	63.6	1.000
Normalized position and partial FA improvement	2	28.6	1	25.0	3	27.3	
No fibers position or FA improvement	1	14.3	0	0.0	1	0.8	
**Preoperative pattern IV (infiltration)**	11	78.6	1	16.7	12	60.0	0.018*
**Postoperative**							
Normalized position and anisotropy	5	45.5	1	100.0	6	50.0	1.000
Normalized position and partial FA improvement	4	36.4	0	0.0	4	33.3	
No fibers position or FA improvement	2	18.1	0	0.0	2	16.7	
**Preoperative pattern V (destruction)**	4	28.6	0	0.0	4	20.0	0.267
No identifiable fiber tracts on FA or directional color maps	4	100.0	0	0.0	4	100.0	

*No* number

*Significant at *p* <0.05

^a^Fisher exact/Fisher-Freeman-Halton exact test

We proceeded to assess the association between tumor grade and each of tumor pattern, surgical excision and postoperative outcomes and imaging (Table 3). A significantly higher percentage of high-grade tumors was removed subtotally compared to the low-grade tumors (78.6% vs. 16.7%, *p* = 0.018). No significant association was detected between tumor grade and postoperative clinical outcome (all *p* >0.05; Table 3).

Table 3 summarizes these patterns and the detected postoperative DTI changes for each pattern.

Pattern I (not affected) was detected in 2 (10%) cases [all of low-grade group (33.3 %)]. Displacement pattern appeared preoperatively in 15 cases (5 low-grade and 10 high-grade tumors). Postoperatively, there was normalization of position and anisotropy in 12 patients (80%) [5 low-grade (100%) and 7 high-grade patients (70%)], while normalized position and partial improvement of FA was seen in 3 patients (20%) [all of high-grade tumors (30%)].

The edematous pattern was detected preoperatively in 11 (55%) cases [4 low-grade and 7 high-grade tumors]. Postoperatively, imaging showed normalization of position and anisotropy in 7 patients (63.6%) [3 low-grade (75%) and 4 high-grade patients (57.1%)]; normalized position and partial improvement of FA was seen in 3 patients (27.3%) [1 low-grade (25%) and 2 high-grade (28.6%)]; and no fibers position or FA improvement was detected in 1 patient (0.8%) [with high-grade tumor (14.3%)].

The infiltration pattern was detected preoperatively in 12 (60%) cases [1 low-grade case and 11 high-grade tumors]. After surgery, imaging showed normalization of position and anisotropy in 6 patients (50%) [1 low-grade (100%) and 5 high-grade (45.5 %)]; normalized position and partial improvement of FA was seen in 4 patients (33.3%) [all had high-grade tumors (36.4%)]; and no fibers position or FA improvement was detected in 2 patients (16.7%) [of high-grade tumor (18.1%)].

The disruption pattern was detected preoperatively in 4 (20%) cases [all had high-grade tumors (28.6 %)]. Postoperatively, imaging showed no identifiable fiber tracts on FA or directional color maps.

**Fig. 8:**
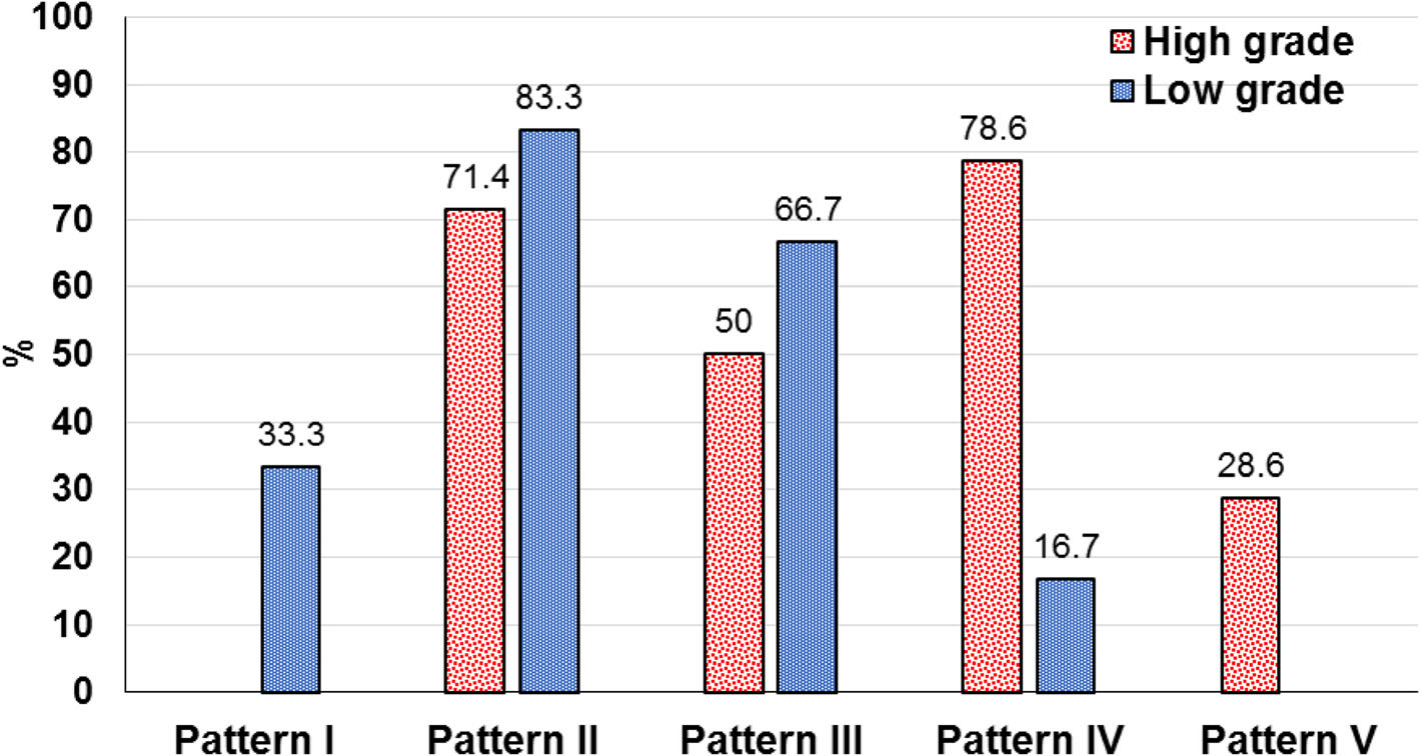
Pattern of brain tumors on imaging

Preoperative imaging showed a higher percentage of infiltration and disruption patterns in the high-grade group, with statistically significant difference in infiltration pattern (78.6% vs. 16.7%, *p* = 0.018; Fig. 8). After surgery, we compared the patterns of tract involvement with the postoperative DTI and tractography. No significant association was detected between postoperative DTI and tractography with tumor grade (all *p* >0.05) (Table 3).

There was a significant association between the pattern of WM tract involvement on preoperative DTI and the postoperative DTI findings, as all cases with disruption pattern showed no identifiable fiber tracts on FA or directional color maps (*p*<0.001; Table 4).

**Table 4:** Association between preoperative DTI patterns and postoperative DTI findings

Postoperative DTI	Preoperative DTI patterns										***p***^**a**^
	I (***n*** = 2)		II (***n*** = 15)		III (***n*** = 11)		IV (***n*** = 12)		V (***n*** = 4)		
	No	%	No	%	No	%	No	%	No	%	
Normalized position and anisotropy	2	100.0	13	86.7	7	63.6	6	50.0	0	0.0	<0.001*
Normalized position and partial FA improvement	0	0.0	2	13.3	3	27.3	4	33.3	0	0.0	
No fibers position or FA improvement	0	0.0	0	0.0	1	9.1	2	16.7	0	0.0	
No identifiable fiber tracts on FA or directional color maps	0	0.0	0	0.0	0	0.0	0	0.0	4	100.0	

*No* number

*Significant at *p* <0.05

^a^Fisher exact/Fisher-Freeman-Halton exact test

## Discussion

Brain tumors may alter WM tracts by displacement, infiltration, or disruption. Precise depiction of the relationship of a brain tumor to its surrounding WM tracts is of crucial importance to plan surgery and determine the extent of tumor resection. The use of DTI tractography can contribute substantially to preoperative planning by confirmation of integrity and location of displaced WM tracts. If preoperative imaging demonstrated that a WM tract was intact but displaced by tumor to a new location (pattern II), surgical approach can be adapted to preserve the displaced tract during resection. Meanwhile, if specific WM tracts were shown by imaging to be disrupted by tumor (pattern V), gross total resection can be attempted without concern for these WM tracts [9, 11].

This prospective study analyzed the impact of DTI tractography in preoperative planning and predicting surgical outcome in patients with gliomas. Twenty patients with glioma were included.

In the present study, preoperative DTI showed non-affection of WM tracts, displacement, edema, infiltration, and disruption in 10%, 75%, 55%, 60%, and 20% of cases, respectively. The frequencies of detected patterns differ than those reported by Zhukov et al. [12], Dubey et al. [13], and Khan et al. [10]. Zhukov et al. [12] identified DTI patterns in 29 patients with brain tumor pathologies of different grades. They detected intact pyramidal tract in 41.1%, displacement in 24.1%, and infiltration in 34.5% of cases. Dubey et al. [13] studied 34 patients with brain tumor pathologies of different grades. They observed displacement in 52.9%, invasion in 32.3%, and disruption in 11.7% of patients. Khan et al. [10] reported in a series of 128 patients with brain gliomas and metastasis that displacement, infiltration, and disruption were detected in 32.8%, 25%, and 42.2% of cases, respectively. These differences could be attributed to variations in tumor type, grade and studied WM tracts.

Displacement and infiltration of the WM tract were detected in higher percentage of high-grade gliomas compared to low-grade tumors, though the difference did not reach statistical significance except in infiltration pattern (*p* = 0.018). These findings are in accordance with previous studies which concluded that high-grade tumors are associated with displacement or destruction of WM fiber tracts [10, 12, 13].

The extent of resection was planned according to the degree of involvement of WM tract, where complete resection was performed only in the absence of infiltration. In patients who had tumors infiltrating the WM tracts, subtotal resection was only achievable. This method of planning the surgical procedure was stated by previous studies [13–17]. Previous studies showed that the use of DTI resulted in change of the surgical approach in 16 to 47% of patients and a change in the extent of resection in 64 to 80% [10, 16, 17].

The incorporation of DTI and tractography has been associated with improvement of neurological outcome. Postoperative neurological examination during follow-up showed that cognitive function, motor power, and vision deteriorated after surgery in 3 (15%), 2 (10%), and 1 (5%) patients, respectively. Improvement in motor power was observed in 7 (35%) patients. Overall, 70% of patients had no neurological deficit after surgery. This percentage is slightly lower than those reported by previous studies which ranged from 76 to 88.9% [10, 15–17].

We were unable to evaluate the association between the pattern of WM tract involvement and neurological outcome after surgery due to the presence of combined patterns in patients. However, Khan et al. [10] assessed this relationship and found that improvement was associated with displacement while deterioration was more frequent with disruption pattern, though no statistical significance was elicited.

Comparison of pre- and postoperative DTI in our series of patients demonstrated postoperative improvement in displaced and edematous patterns, while infiltrative and disruptive patterns showed higher percentage of cases without normalization of fibers position or FA improvement. This improvement in parameters of DTI after surgery is attributed to the considerable reorganization of brain structures which may occur following surgical resection. Such changes can be detected using DTI as reported in the literature [18–22].

Normalization of position and FA after surgery was observed in all low-grade cases, except for one case with displaced pattern in which only partial FA improvement occurred. Normalization of position and partial FA improvement occurred at high-grade and—to less extent—low-grade lesions. The lack of fiber tract position or FA improvement was confined to high-grade lesions. However, the postoperative DTI and tractography changes were not significantly associated with tumor grade.

Our results indicated that reorganization of tracts is more likely to occur in tumors with displaced or edematous patterns compared to the infiltrated and disruptive patterns. There is a paucity of studies which evaluated the potential effect of tumor grade or preoperative DTI pattern on postoperative DTI changes.

Though DTI demonstrated its importance and utility in management of brain tumors, the technique still suffers from some limitations. DTI is a user-defined process. Consequently, the results can vary depending on the chosen parameters such as FA threshold, angular threshold, step length, and numbers of sampling in a voxel length. Moreover, tracked volumes may differ according to the size and locations of the seed regions of interest (ROIs).

Other important limitation of low number of cases is the difficulty to follow and bring the same patients before and after operation; moreover, some of them suffer neurological insult, in addition to COVID-19 pandemic sequelae .Also our study was designed with inclusion criteria including patients harbored different grads of gliomas *only* and not other brain space occupying lesions .

## Conclusion

DTI provides better assessment of the effect of cerebral tumors on WM tracts compared to conventional MRI. This effect may take the form of one or more of five patterns. The provided data were helpful in designing the surgical approach and extent of tumor resection. The preoperative pattern of WM tract involvement was significantly associated with the postoperative DTI changes, which may be reflected in better clinical neurological outcome. Tumor grade may affect postoperative DTI changes with lack of fiber tract position or FA improvement being confined to high-grade lesions.

## Recommendation

Future prospective studies with larger sample size should be conducted to confirm these promising findings. The assessment of the potential effect of tumor grade on DTI patterns is recommended.

## Data Availability

The datasets generated or analyzed during current study are not publicly available as the license taken for the current study only, not public. But the data are available on reasonable request from corresponding author.

## Abbreviations

3D: Three dimensional
ADC: Apparent diffusion coefficient
CST: Cortico-spinal tract
DTI: Diffusion tensor imaging
DWI: Diffusion-weighted imaging
FLAIR: Fluid-attenuated inversion recovery
FT: Fiber tractography
GBM: Glioblastoma multiform
Ilf: Inferior longitudinal fasciculus
MRI: Magnetic resonance imaging
SLF: Superior longitudinal fasciculus
UF: Uncinate fasciculus
WM: White matter
FA: Fractional anisotropy
WHO: World Health Organization
